# COVID-19 Vaccination in Adults: Results from the Tanzania HIV Impact Survey 2022–2023

**DOI:** 10.3390/vaccines13121185

**Published:** 2025-11-24

**Authors:** Damian Jeremia Damian, Stephano G. Cosmas, Alice Wang, Magreth Kagashe, Aisha Haji, Jocelyn Rwehumbiza, Fahima Issa, Ahmed Khatib, Emilian Karugendo, Geofrey Mchau, Samuel Sumba, Rebecca Laws, Mary Mayige, Nicolas Schaad, Jerome Kamwela, Faki Faki, Deogratias Morice Kakiziba, Nyambura Moremi, Mahesh Swaminathan, Sarah E. Porter, Prosper Pendo

**Affiliations:** 1 U.S. Centers for Disease Control and Prevention, P.O. Box 9123 Dar es Salaam, Tanzania; 2 National Bureau of Statistics, P.O. Box 2683 Dodoma, Tanzania; 3 Tanzania Commission for AIDS, P.O. Box 2904 Dodoma, Tanzania; 4 Office of the Chief Government Statistician, P.O. Box 2321 Zanzibar, Tanzania; 5 Zanzibar AIDS Commission, P.O. Box 2820 Zanzibar, Tanzania; 6 U.S. Centers for Disease Control and Prevention, Atlanta, GA 30329-4018, USA; 7 National Institute for Medical Research, P.O. Box 9653 Dar es Salaam, Tanzania; 8 Zanzibar Integrated HIV, Hepatitis, Tuberculosis and Leprosy Programme, P.O. Box 1300 Zanzibar, Tanzania; 9 ICAP Tanzania, P.O. Box 80214 Dar es Salaam, Tanzania; dmk2209@cumc.columbia.edu; 10 National Public Health Laboratory, P.O. Box 9083 Dar es Salaam, Tanzania; 11 National AIDS, STI, and Hepatitis Control Programme, P.O. Box 784 Dodoma, Tanzania

**Keywords:** COVID-19, COVID-19 vaccines, vaccination hesitancy, chronic conditions, Tanzania

## Abstract

**Background**: During the COVID-19 pandemic, vaccine hesitancy was high in Tanzania. Reasons for vaccine hesitancy included skepticism about vaccine safety and efficacy. Nonetheless, by March 2023, following concerted efforts, Tanzania reported reaching a national COVID-19 vaccination coverage of 52.5% among adults. We analyzed COVID-19 vaccination status and perceived vaccine safety among adults aged ≥ 18 years using data from the Tanzania HIV Impact Survey (THIS) 2022–2023. **Methods**: The THIS 2022–2023 was a nationally representative, cross-sectional household survey with a stratified two-stage cluster design. Descriptive analyses were conducted to determine the proportion of adults who self-reported receiving the COVID-19 vaccine andcompleting the primary vaccine series, and to assess vaccine safety perceptions. Analyses were weighted for non-response and accounted for complex survey design. **Results**: Of 32,777 adults, 20.0% (95% confidence interval (CI): 18.9–21.1) reported receiving more than one COVID-19 vaccine dose. Of those, 82.9% (95% CI: 81.3–84.5) completed the primary vaccination series. The proportion of adults who reported vaccination increased with age from 12.5% among 18–24 year olds to 29.0% among those aged 55–64 years. Among adults living with HIV (ALHIV), 49.6% (95% CI: 47.1–52.1) were vaccinated, and vaccination rates increased with longer duration on antiretroviral therapy. Adults who perceived the vaccines as safe (27.9%, 95% CI: 26.4–29.3) were 10 times more likely to report being vaccinated than those who perceived the vaccines as “not at all safe” (2.7%, 95% CI: 2.0–3.6). **Conclusions**: The COVID-19 vaccination coverage in Tanzania among adults ≥ 18 years as measured through the THIS 2022–2023 was less than half the national COVID-19 vaccine coverage reported in March 2023. ALHIV were more likely than the general population to be vaccinated. The substantial difference in vaccination rates between those who perceived the vaccines as safe versus unsafe highlights the importance of safety perception for vaccine uptake.

## 1. Introduction

The first case of COVID-19 in the United Republic of Tanzania was reported on 16 March 2020, and many public health measures recommended by the World Health Organization (WHO) were implemented immediately [1,2,3,4]. In June 2021, Tanzania joined the COVAX initiative and authorized COVID-19 vaccines for use [2,5]. Tanzania received the first shipment of COVID-19 vaccines in July 2021 and launched the country’s first vaccination campaign in August 2021 [6]. The original vaccination policy prioritized groups including adults with chronic medical conditions, frontline health care workers, and individuals aged > 45 years. In February 2022, the vaccination policy was expanded to the general population aged ≥ 18 years [7,8,9]. Persons aged < 18 years were also eligible under special circumstances, including to comply with foreign travel requirements [2].

In Tanzania, COVID-19 vaccines were initially met with safety and efficacy concerns, fueled in part by intense debate on social media and other media channels [7,10]. At the beginning of 2022, only 3.2% of the Tanzanian population was reported to be vaccinated, and heightened political commitment at the national, regional, and district levels was focused on increasing uptake [11,12]. Tanzania implemented a mass education and community engagement campaign and made vaccines available at health facilities, community events, workplaces, and households to increase access [13]. The President’s Emergency Plan for AIDS Relief (PEPFAR) leveraged HIV service support during the COVID-19 response in Tanzania, from providing human resources for surge capacity, to vaccine delivery in HIV clinics, to the use of data systems for COVID-19 data management and surveillance [14]. By March 2023, COVID-19 vaccination coverage was reported to have reached 52.5%, and Tanzania was lauded by the WHO for achieving such a rapid turnaround [11,12]. By the end of 2023, a total of 39.4 million COVID-19 vaccine doses were administered [15]. Tanzania received the Johnson and Johnson/Janssen, Sinopharm, Pfizer/BioNTech, and Moderna vaccines via the COVAX initiative, and Sinopharm and Sinovac vaccines through bilateral agreements [1].

Studies showed high levels of COVID-19 vaccine hesitancy among health care workers, other frontline workers, and the general population in Tanzania [5,10,16,17,18,19,20,21]. A study in western Tanzania found 62.0% of health care workers were hesitant about COVID-19 vaccines, due to a perceived lack of reliable information regarding efficacy and safety [16]. Over 80% of frontline workers in the Dar es Salaam and Dodoma regions reported exposure to manipulated, fabricated, or misleading content about COVID-19 vaccines [17]. In rural eastern Tanzania, belief in alternative medicines (38.1%) and fear of side effects (33.7%) were the main reasons for COVID-19 vaccine refusal [1]. A hospital- and community-based survey identified three main reasons for COVID-19 vaccine hesitancy: fear of side effects, spiritual or religious views, and belief in alternative local remedies and precautions [7]. A community-based study in eight regions of Tanzania and Zanzibar found that only 54.6% of adults believed COVID-19 vaccines to be both safe and effective, and that those who were confident in the vaccines were three times more likely to have been vaccinated [19].

We describe COVID-19 vaccination among adults aged ≥ 18 years by sociodemographic and clinical characteristics, as measured through a cross-sectional survey conducted at the same time as the vaccine delivery efforts described above. The objectives of the analysis were to quantitatively measure COVID-19 vaccination and perceptions of COVID-19 vaccine safety and efficacy among adults in Tanzania.

## 2. Materials and Methods

### 2.1. Study Design and Participants

We used data from the Tanzania HIV Impact Survey (THIS) 2022–2023, conducted between November 2022 and March 2023. The THIS 2022–2023 was a nationally representative, cross-sectional, population-based survey of adults aged ≥ 15 years from all regions of Tanzania. The survey employed a multi-stage approach using a two-stage stratified cluster design; the methodology has been previously described elsewhere [22]. Adults were administered a survey that included questions on COVID-19 vaccination and safety perceptions, HIV status, duration of antiretroviral therapy (ART), and chronic conditions. HIV status was determined by HIV rapid testing at the household level and confirmatory testing at satellite and main laboratories. To ascertain COVID-19 vaccination history, participants were asked whether they had ever received a COVID-19 vaccine. Those that responded that they had received a COVID-19 vaccine were asked if they could produce their vaccination record. While the THIS 2022–2023 included adults aged ≥ 15 years, this analysis was limited to adults aged ≥ 18 years to account for vaccination eligibility based on Tanzania’s COVID-19 guidelines. In addition, we excluded participants who responded “do not know” or refused to the COVID-19 vaccination uptake question.

### 2.2. Variable Definitions

The primary outcome variable was receiving at least one dose of COVID-19 vaccine, ascertained through self-report regardless of whether participants produced a vaccination record or not. Unless otherwise stated, “vaccinated” refers to receiving at least one dose of a COVID-19 vaccine. Adults were considered to have completed a primary COVID-19 vaccine series if they reported receiving one dose of a single-dose vaccine or two doses on different days of an mRNA or protein-based vaccine, regardless of the time interval. The secondary outcome was perceived COVID-19 vaccine safety. The perceived safety of COVID-19 vaccines was categorized into three groups: safe (i.e., adults responded a little safe, moderately safe, and very safe to the vaccine safety question), not safe, and do not know or refused. Exposure variables included demographic variables such as age, sex, education, marital status, wealth quintile, region, and area of residence. Other variables included confirmed HIV status through HIV testing using the national algorithm, duration on ART for those who self-reported being HIV-positive, and self-reported chronic conditions.

### 2.3. Statistical Analyses

Data were analyzed in Stata version 17 (StataCorp. 2022. Stata Statistical Software: Release 17. College Station, TX, USA: StataCorp LLC). Descriptive analyses were conducted to describe the sociodemographic characteristics of the population, to determine COVID-19 vaccination by area of residence (i.e., geography) and clinical characteristics, and vaccination in relation to perceptions of safety. Analyses were weighted for non-response and accounted for the complex survey design. Jackknife variance estimation was used to generate 95% confidence intervals (CI).

## 3. Results

### 3.1. Sociodemographic Characteristics of the Study Population

Among 35,957 adults from THIS 2022–2023, 32,805 were adults aged ≥ 18 years. Of these, 32,477 (99.0%) responded to questions on COVID-19 vaccination, which excluded those who responded, “do not know” or “refused”. Among these adults aged ≥ 18 years, 52.5% were female, 60.9% resided in rural areas, 53.3% were aged 18–34 years, 57.9% were educated to at least primary level, and 62.5% were either married or living with a partner (Table 1).

### 3.2. COVID-19 Vaccination Status

Among adults aged ≥ 18 years, 7058 (20.0% [95% CI: 18.9–21.1]) reported that they had received at least one COVID-19 vaccine dose (Table 1). Of those reporting vaccination, 57.3% (55.2–59.4) provided a vaccination record. Among those vaccinated, 61.2% (95% CI: 58.1–64.1) reported that they received the single dose Johnson and Johnson/Janssen vaccine (Janssen Pharmaceuticals, Raritan, NJ, USA). Of those who received two-dose COVID-19 vaccines, 84.2% (95% CI: 81.0–87.1) completed the vaccine series. Overall, 82.9% (95% CI: 81.3–84.5) of those vaccinated reported completing the primary vaccine series (Table 2).

### 3.3. COVID-19 Vaccine Uptake by Sociodemographic Characteristics

**Figure 1:**
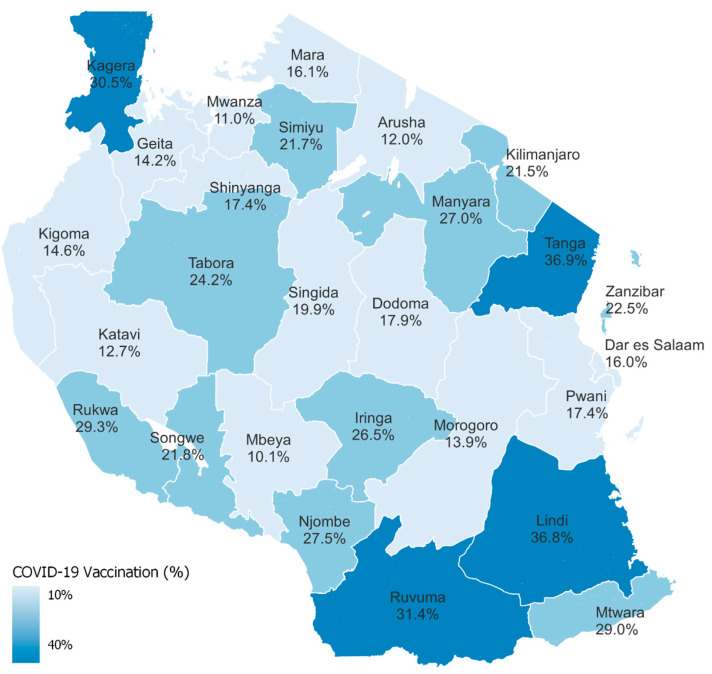
Proportion of adults ≥ 18 years who self-reported receiving at least one COVID-19 vaccination by region in Tanzania, 2022–2023.

A higher proportion of women (22.6%, 95% CI: 21.2–24.0) reported being vaccinated than men (17.2%, 95% CI: 16.1–18.3). The uptake of COVID-19 vaccines was lower among younger adults than older adults, ranging from 12.5% (95% CI: 11.4–13.6) among adults aged 18–24 years to 29.0% (95% CI: 26.7–31.6) among those aged 55–64 years. Adults who completed secondary education reported a lower vaccine uptake (15.3%, 95% CI: 14.2–16.6) compared to other educational level categories. People who had never been married were less likely to report COVID-19 vaccination (10.9%, 95% CI: 9.9–12.0) compared to those who were married or living together (22.0%, 95% CI: 20.6–23.5), divorced or separated (22.4%, 95% CI: 20.6–24.3), or widowed (28.3%, 95% CI: 25.8–30.9). Adults in the higher wealth quintiles were less likely to report being vaccinated: 15% (95% CI: 14.1–17.3) of those in the highest quintile reported being vaccinated, compared to 22.1% (95% CI: 20.0–24.3) of those in the lowest quintile. The reported vaccination uptake was higher in rural areas (22.9%, 95% CI: 21.3–24.7) than in urban areas (15.5%, 95% CI: 14.3–16.7) (Table 1). There was substantial regional variation in reported COVID-19 vaccination by region, ranging from 10.1% in Mbeya to 36.9% in Tanga (Figure 1).

### 3.4. COVID-19 Vaccine Uptake by Clinical Characteristics

A higher proportion of adults living with HIV (49.6%, 95% CI: 47.1–52.1), in particular those who reported being on ART for ≥ 10 years, (65.2%, 95% CI: 59.1–70.9) reported being vaccinated than HIV-negative adults. Reported COVID-19 vaccination was also higher among adults with other chronic conditions, including diabetes (32.4%, 95% CI: 27.0–38.3), hypertension (30.9%, 95% CI: 27.5–34.5), and heart disease (29.8%, 95% CI: 23.7–36.7), compared to those who had no chronic condition (19.5%, 95% CI: 18.4–20.7) (Table 3).

### 3.5. Perceived Safety of COVID-19 Vaccines

**Figure 2:**
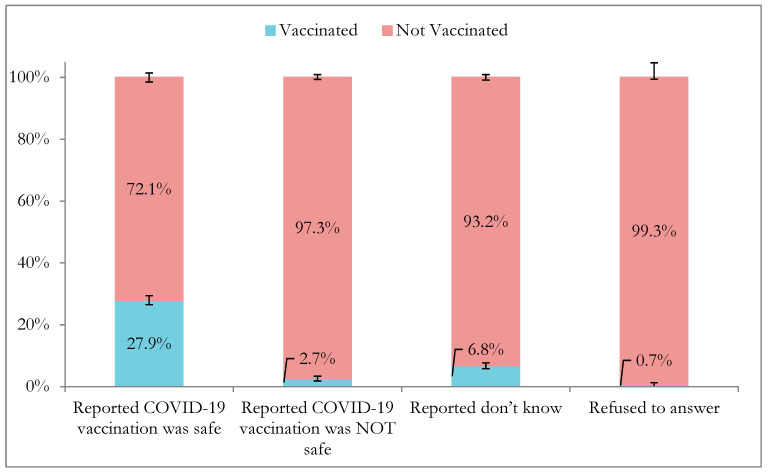
Self-reported COVID-19 vaccination status by perceived safety of COVID-19 vaccine among adults ≥ 18 years in Tanzania, 2022–2023 (*N* = 32,477). Lines indicate 95% confidence intervals. Reported COVID-19 vaccination was “safe” includes adults who reported “very safe”, “moderately safe”, and “a little safe”.

Among adults, 65.5% perceived COVID-19 vaccines to be safe (i.e., 37.3% “very safe”, 17.6% “moderately safe”, and 10.7% “a little safe”,) and 13.3% felt that they were “not at all safe”. Adults who perceived the vaccines as safe (27.9%, 95% CI: 26.4–29.3) were over 10 times more likely to report being vaccinated than those who perceived the vaccines as “not at all safe” (2.7%, 95% CI: 2.0–3.6) (Figure 2). The reported vaccine uptake also increased by the level of perceived safety, from 9.0% (95% CI: 7.6–10.6) among those who felt that vaccines were “a little safe” to 13.5% (95% CI: 12.1–15.1) among those who felt that they were “moderately safe”, and finally to 40.0% (95% CI: 38.1–42.0) among those who felt they were “very safe” (Table 4).

## 4. Discussion

Tanzania’s COVID-19 vaccination campaign was reported to have reached 34.4 million eligible individuals, with 43.1% coverage at the beginning of the THIS 2022–2023 data collection period and 52.3% coverage by the end of THIS 2022–2023 [11,15]. Self-reported COVID-19 vaccination among adults ≥ 18 years who participated in the THIS 2022–2023 was less than half of the COVID-19 vaccine coverage rates reported by national authorities. There may be multiple reasons for the discrepancy between reported national COVID-19 vaccination coverage and survey findings. To begin with, vaccine coverage estimates calculated using the administrative method, as the figures reported by national authorities were, can be biased due to inaccurate numerators or denominators [23,24]. The vaccine coverage rates reported by national authorities used the population of individuals ≥ 18 years from a 2022 projection based on the 2012 Tanzania census as a denominator. However, this projected figure (30,740,928) did not differ substantially from the actual figure from the 2022 Tanzania census (31,483,925) and this small difference could not alone account for the extent of the discrepancy between reported coverage rates and THIS 2022–2023 results [25]. Inconsistent record-keeping during the vaccination campaign and non-standardized data management practices across the many local and international partners assisting with Tanzania’s COVID-19 vaccination response may also have introduced inaccuracies on the numerator side of reported COVID-19 vaccination coverage [5]. On the other hand, self-reported COVID-19 vaccination in the THIS 2022–2023 may have been subject to information and recall bias, similar to other vaccine coverage surveys [24]. Finally, the analysis was conducted with a slightly different comparison period than the national COVID-19 vaccination report.

People with chronic conditions were a high priority for the national vaccination campaign, and as expected, COVID-19 vaccination rates were found to be slightly higher among adults with other chronic conditions than among the general adult population [2]. To answer the first analysis objective, the COVID-19 vaccination coverage among PLHIV was more than twice the coverage of all adults, which was likely due to COVID-19 vaccination integration with routine HIV care and treatment services and COVID-19 vaccine offered to all people during outpatient services [6,26]. It is also important to note that differences in vaccination may have been a result of differing service delivery approaches in different settings. Urban populations typically accessed COVID-19 vaccines at health facilities, whereas rural populations were primarily reached through outreach (i.e., house-to-house) campaigns and at temporary vaccination points in the community (e.g., markets, places of worship, sporting events [27]. These results suggest that employing tailored vaccination campaigns to complement facility-based vaccination in urban areas is important for maximizing vaccination coverage in this setting.

Only 65.5% of adults agreed that COVID-19 vaccines were “very safe”. This is consistent with findings from other studies among the general adult population in Tanzania [1,10,18,19]. With respect to the second analysis objective, those who reported that COVID-19 vaccines were safe were over ten times more likely to have been vaccinated compared to those who did not perceive vaccines to be safe, which was also consistent with findings from other studies [19]. These results demonstrate the importance of positive perceptions of vaccine safety and may be an important catalyst for vaccination. The implementation of risk communication and vaccine sensitization interventions, as well as community engagement efforts to increase trust around COVID-19 vaccination remain vital for reducing vaccine hesitancy and improving vaccination rates [28,29].

A major strength of this analysis was that it was conducted on a robust dataset from a large-scale, nationally and sub-nationally representative survey with a rigorous and widely used methodology. A major limitation was that COVID-19 vaccination status was self-reported, and only a little over half of participants who reported being vaccinated produced a vaccination record for verification. Tanzania does not have a consolidated national vaccine registry, and it was also not possible to independently confirm COVID-19 vaccination status.

## 5. Conclusions

The COVID-19 vaccination coverage in Tanzania among adults ≥ 18 years as measured through the THIS 2022–2023 was 20.0%, equivalent to less than half the reported national COVID-19 vaccine coverage around the same time as the survey. Although most adults perceived COVID-19 vaccination to be safe, over a quarter were not vaccinated. Adults living with HIV were much more likely to have reported COVID-19 vaccination. The substantial difference in vaccination rates between those who perceived the vaccines to be safe versus unsafe, and the fact that nearly all adults who perceived that COVID-19 vaccination was not safe were not vaccinated, highlight the importance of safety perception for vaccine uptake.

## Figures and Tables

**Table 1:** Socio-demographic characteristics and self-reported COVID-19 vaccination among adults ≥ 18 years in Tanzania, 2022–2023 (*N* = 32,477).

Variables	Total (%)	% Vaccinated (95% CI *)
Total	32,477 (100)	20.0 (18.9–21.1)
**Residence**		
Urban	11,058 (39.1)	15.5 (14.3–16.7)
Rural	21,419 (60.9)	22.9 (21.3–24.7)
**Area**		
Tanzania mainland	30,657 (96.3)	19.9 (18.8–21.1)
Zanzibar	1820 (3.7)	22.5 (18.5–27.1)
**Sex**		
Male	13,796 (47.5)	17.2 (16.1–18.3)
Female	18,681 (52.5)	22.6 (21.2–24.0)
**Age, years**		
18–24	7306 (25.9)	12.5 (11.4–13.6)
25–34	8367 (27.4)	18.2 (16.9–19.6)
35–44	6252 (18.7)	22.5 (21.0–24.2)
45–54	4705 (13.1)	25.3 (23.2–27.5)
55–64	3006 (7.6)	29.0 (26.7–31.6)
65+	2841 (7.3)	28.0 (25.5–30.7)
**Highest Education Level (*N* = 32,444)**		
No education	5283 (14.4)	20.9 (18.9–23.1)
Primary	19,259 (57.9)	21.5 (20.1–22.9)
Secondary	6837 (23.6)	15.3 (14.2–16.6)
Post-secondary	1065 (4.1)	22.7 (18.9–27.1)
**Marital Status (*N* = 32,442)**		
Never married	5853 (21.8)	10.9 (9.9–12.0)
Married or living together	20,946 (62.5)	22.0 (20.6–23.5)
Divorced or separated	3192 (9.6)	22.4 (20.6–24.3)
Widowed	2451 (6.1)	28.3 (25.8–30.9)
**Wealth Quintile (*N* = 32,464)**		
Lowest	7093 (21.0)	22.1 (20.0–24.3)
Second	7340 (20.1)	23.8 (21.6–26.2)
Middle	7150 (20.5)	20.2 (18.4–22.2)
Fourth	5754 (19.1)	17.9 (16.3–19.6)
Highest	5127 (19.3)	15.6 (14.1–17.3)

* CI—Confidence Interval.

**Table 2:** Prevalence of vaccine clinical characteristics among adults aged ≥ 18 years who self-reported receiving at least one COVID-19 vaccine dose in Tanzania, 2022–2023 (*N* = 7058).

Variable	n	Weighted % (95% CI)
**Provided vaccination record**		
Yes	4120	57.3 (55.2–59.4)
No	2398	42.7 (40.6–44.8)
**Number of vaccine doses received, of those vaccinated**		
1	5073	71.5 (68.6–74.3)
2	1895	27.0 (24.3–29.9)
3+	40	0.8 (0.5–1.1)
Refused/Do not know	50	0.7 (0.5–1.1)
**Brand of first vaccine dose**		
Johnson and Johnson/Janssen	4311	61.2 (58.1–64.1)
Pfizer (Pfizer, New York, NY, USA)	664	9.6 (8.12–11.3)
Sinopharm (Sinopharm, Beijing, China)	1017	14.8 (13.1–16.7)
Moderna (Moderna, MA, USA)	44	0.7 (0.3–1.8)
Sinovac (Sinovac, Beijing, China)	74	0.8 (0.6–1.1)
Refused/Do not know which vaccine was received/other	948	12.9 (11.5–14.5)
**Completed 2-dose vaccine series (*N* = 1798)**		
Yes	1520	84.2 (81.0–87.1)
No	278	15.7 (12.9–19.0)
Completed primary vaccine series *		
Yes	5831	83.0 (81.3–84.6)
No	278	4.1 (3.4–4.9)
Refused/Do not know which vaccine was received	949	13.0 (11.6–14.5)

* Adults are considered to have completed a primary series if they received at least one dose of a single-dose vaccine or two doses on different days (regardless of time interval) of either an mRNA or a protein-based series.

**Table 3:** Self-reported COVID-19 vaccination among adults ≥ 18 years by clinical characteristics in Tanzania, 2022–2023.

Variables	Total (%)	% Vaccinated (95% CI)
**HIV Status (*N* = 32,477)**		
Positive	1823 (4.6)	49.6 (47.1–52.1)
Negative	28,564 (88.1)	18.7 (17.6–20.0)
Not tested	2090 (7.4)	16.7 (14.8–21.2)
Other chronic conditions *		
Diabetes	352 (1.1)	32.4 (27.0–38.3)
Hypertension	1307 (3.6)	30.9 (27.5–34.5)
Heart disease	319 (0.8)	29.8 (23.7–36.7)
Kidney disease	138 (0.4)	20.6 (15.0–27.6)
Cancer or tumor	306 (0.8)	24.8 (19.0–21.2)
Lung disease	233 (0.6)	27.3 (20.9–34.7)
Mental health condition	78 (0.2)	24.5 (14.5–38.2)
No chronic conditions	29,814 (92.4)	19.5 (18.4–20.7)
Number of years since initiating ART ** (*N* = 1424)		
Less than 1 year	128 (9.7)	46.0 (35.6–57.4)
1 year to less than 5	482 (32.9)	53.7 (48.6–58.6)
5 years to less than 10	431 (30.3)	55.9 (50.0–61.7)
10 years or more	383 (27.1)	65.2 (59.1–70.9)

ART = antiretroviral therapy; * Among those who provided responses; ** Among those who self-reported being HIV positive; CI = Confidence Interval.

**Table 4:** Self-reported COVID-19 vaccination among adults ≥ 18 years by perceived vaccine safety in Tanzania, 2022–2023 (*N* = 32,477).

Variable	Total (%)	% Vaccinated (95% CI)
**Perceived COVID-19 vaccine safety**		
Safe	21,338 (65.5)	27.9 (26.4–29.3)
Very safe	12,349 (37.3)	40.0 (38.1–42.0)
Moderately safe	5616 (17.6)	13.5 (12.1–15.1)
A little safe	3373 (10.7)	9.0 (7.6–10.6)
Not safe	4089 (13.3)	2.7 (2.0–3.6)
Do not know	6825 (20.5)	6.8 (5.9–7.8)
Refused	225 (0.7)	0.7 (0.1–5.4)

CI = Confidence Interval.

## Data Availability

The original data presented in the study are openly available at www.nbs.go.tz, accessed on 1 July 2025.

## Abbreviations

The following abbreviations are used in this manuscript:

ALHIV	Adults living with HIV
ART	Antiretroviral therapy
CDC	Centers for Disease Control and Prevention
CI	Confidence interval
THIS	Tanzania HIV Impact Survey
WHO	World Health Organization
